# The Airway Epithelial Barrier in Childhood Asthma: A Central Player in Glucocorticoid Therapy, Resistance, and Severe Asthma

**DOI:** 10.1002/pdi3.70056

**Published:** 2026-05-26

**Authors:** Tangqiaochu Gan, Zhou Fu, Chao Niu

**Affiliations:** ^1^ Department of Respiratory Medicine Children's Hospital of Chongqing Medical University National Clinical Research Center for Child Health and Disorders, Ministry of Education Key Laboratory of Child Development and Disorders, Chongqing Engineering Research Center of Stem Cell Therapy Chongqing China

**Keywords:** airway epithelial barrier, asthma, childhood asthma, glucocorticoid resistance, glucocorticoids, severe asthma

## Abstract

Childhood asthma remains a global public health challenge with suboptimal control, despite the availability of inhaled corticosteroids (ICS) as first‐line therapy. Glucocorticoids suppress inflammation and protect the airway epithelial barrier (AEB) via genomic and nongenomic pathways. However, a significant subset of patients, particularly those with severe asthma, exhibits glucocorticoid resistance. Emerging evidence reveals a dual role of glucocorticoids on the AEB: beyond protection, they may inadvertently compromise barrier integrity by inducing epithelial apoptosis and dysfunction. This review synthesizes current knowledge on glucocorticoid mechanisms in asthma, focusing on the AEB as a critical interface between therapeutic efficacy and treatment failure. We show that AEB impairment serves as one of the key mechanisms underpinning glucocorticoid resistance and the progression to severe asthma, with heightened relevance in children due to the unique vulnerability of their developing airways. Furthermore, we examine how infections, nutritional factors (e.g., vitamins A and D), and immune maturation intersect with AEB integrity and glucocorticoid responsiveness. By reframing the AEB as both a target and a determinant of glucocorticoid efficacy, this review highlights the urgent need for barrier‐focused strategies to overcome resistance and improve outcomes in childhood asthma.

## 1 Introduction

Bronchial asthma is the most common chronic airway disease in children, imposing a substantial burden on patients, their families, and the society. Surveys in China have shown that the prevalence of asthma among urban children aged 0–14 years was 1.09% in 1990, 1.97% in 2000, and 3.02% in 2010, demonstrating a significant upward trend [1]. However, asthma control in children remains unsatisfactory in China. Studies suggest that approximately 30% of urban children with asthma may not receive a timely diagnosis [2], and over 20% are not controlled satisfactorily [3]. Therefore, early diagnosis, timely treatment, and improved therapeutic efficacy are crucial for enhancing asthma control and reducing the associated disease burden.

**FIGURE 1:**
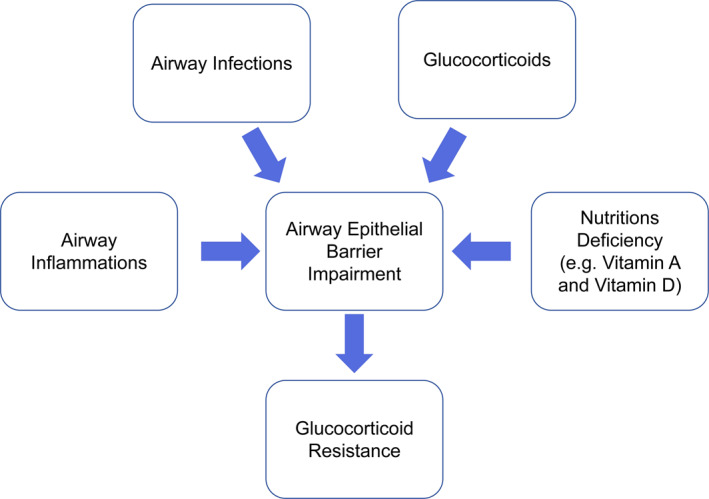
Variables associated with airway epithelial barrier (AEB) impairment that may lead to glucocorticoids resistance addressed in this review. This figure illustrates the multifactorial nature of AEB impairment contributing to glucocorticoid resistance, including infections, nutritional deficiencies, and inflammatory mediators.

The airway epithelial barrier (AEB) is a critical component of the human respiratory tract. Comprising the airway epithelium and the airway surface liquid layer, it plays a vital role in the development and progression of asthma. Current evidence suggests that glucocorticoids control asthma by downregulating airway inflammation, mitigating AEB impairment, and inhibiting airway epithelial remodeling. Inhaled corticosteroids (ICSs), a primary form of glucocorticoid‐based treatment, are the first‐line therapy for asthma. Nevertheless, 5%–10% of patients exhibit poor or absent response to glucocorticoid therapy [4]. Refractory asthma among these cases, excluding cases attributable to poor medication adherence or inadequate inhalation technique, is termed severe asthma [4]. The mechanisms underlying severe asthma are not completely understood but may be closely linked to the multifaceted effects of glucocorticoids in asthma and their potential damaging impact on the AEB [4, 5]. This review summarizes the critical role of AEB in glucocorticoid‐based asthma therapy, glucocorticoid resistance and severe asthma and its influencing factors (Figure 1).

This review posits that preserving or restoring AEB integrity may be as crucial as suppressing inflammation for achieving durable glucocorticoid responses and preventing severe asthma in children.

## 2 The Airway Epithelial Barrier Serves as the Body’s First Line of Defense

The AEB comprises the airway epithelium and the airway surface liquid. The airway epithelium consists of various cell types, including ciliated columnar epithelial cells, goblet cells, club cells, and basal cells [6]. Among these, basal cells are believed to have the capacity to differentiate into other cell types [7]. These cells are interconnected via tight junctions, adherens junctions, and desmosomes, forming a continuous cellular barrier. This structure prevents the invasion of allergens and pathogens into the body, thereby averting airway inflammation and facilitating intercellular communication [7, 8].

Beyond the cellular barrier, the airway epithelium secretes various defensive proteins, including mucins, defensins, and antimicrobial peptides [8], contributing to the formation of the airway surface liquid layer with antibacterial, antiprotease, and antioxidant properties, which further strengthens the defensive function of AEB [7]. Through these physical and chemical barrier effects, the AEB limits the interaction between allergens, pathogens, and immune cells, thereby preventing excessive airway inflammatory responses [6, 7].

## 3 Airway Epithelial Cells are Key Regulators of Airway Inflammation

In addition to its barrier function, the AEB plays a crucial role in regulating airway inflammation. Airway epithelial cells express a variety of pattern recognition receptors (PRRs), such as cytoplasmic NOD‐like receptors (NLRs) and transmembrane toll‐like receptors (TLRs). These receptors respond to damage‐associated molecular patterns (DAMPs) and pathogen‐associated molecular patterns (PAMPs) [9]. Upon recognition of harmful stimuli, airway epithelial cells regulate early inflammatory events and participate in local acute inflammatory responses by transcribing and secreting antimicrobial, proinflammatory proteins, and mucins [10, 11].

Furthermore, airway epithelial cells regulate the recruitment of inflammatory cells—including dendritic cells, T cells, B cells, eosinophils, and neutrophils—to the airways by releasing interleukins (ILs), tumor necrosis factors (TNFs), CXCL8, CCL11, and CCL20 [12, 13, 14, 15, 16, 17]. This process is essential for the regulation and coordination of local immunity.

In the presence of harmless antigens, airway epithelium limits immune responses by inhibiting the activation and function of proinflammatory mediators [7], reducing its own sensitivity and reactivity, and secreting anti‐inflammatory factors to block excessive inflammatory responses [7, 18].

In the presence of harmful pathogens or allergens, antigens not cleared by the mucociliary system can be recognized by airway epithelial cells [12], triggering antigen‐specific immune responses. During viral infections, airway epithelial cells continuously produce interferon beta (IFN‐β) to reduce viral replication and promote infected epithelial cell apoptosis [19]. Similarly, in asthma, airway epithelial cells are one of the major producers of various proinflammatory cytokines and chemokines that drive and sustain airway inflammation [20].

Cytokines such as thymic stromal lymphopoietin (TSLP), IL‐25, and IL‐33, secreted by airway epithelial cells, can promote the differentiation of T cells toward a Th2 phenotype [21]. Additionally, the granulocyte‐macrophage colony‐stimulating factor (GM‐CSF) secreted by these cells supports eosinophil maturation and survival [22, 23]. By producing TSLP, airway epithelial cells can create a local microenvironment that favors and maintains Th2 polarized inflammation [20].

However, in asthma, the above‐mentioned regulatory functions may become disordered, resulting in the AEB becoming a target of airway inflammation.

## 4 AEB Impairment in Asthmatic Airway Inflammation

Asthmatic patients exhibit pathological alterations in the airway epithelium, including the impairment and shedding of ciliated columnar epithelial cells, as well as goblet cell hyperplasia and metaplasia [24, 25]. Some of these changes occur as early as the initial stages of asthma [26]. The increased number and hyper‐secretory state of goblet cells contribute to the dysfunction of the airway surface liquid layer in asthma [27, 28, 29].

Studies have shown that acute wheezing of children is correlated to the compromised epithelial barrier integrity [30]. The structural impairments, the dysfunction of physical and chemical barrier functions, and dysregulated inflammatory response of AEB increase the risk of invasion and sensitization by airway pathogens and allergens. Moreover, impaired epithelium releases autoantigens, further exacerbating the airway inflammation and establishing a vicious cycle between airway inflammation and AEB impairment.

## 5 Glucocorticoids Inhibit Airway Inflammation and Protect the AEB

The prevailing view is that glucocorticoids exert anti‐inflammatory and anti‐asthmatic effects through both genomic and nongenomic mechanisms [31]. The genomic mechanism involves the binding of glucocorticoid to cytoplasmic glucocorticoid receptors (GRs) in target cells, forming a glucocorticoid‐GR complex. This complex translocates to the nucleus, where it regulates the transcription of specific genes—either by direct DNA binding or by inactivating transcription factors—to modulate inflammatory responses. Nongenomic effects include nonspecific interactions with the cell membrane, rapid signaling mediated by cytoplasmic GRs, or specific interactions via membrane‐bound GRs [31]. Through these genomic and nongenomic mechanisms, glucocorticoids inhibit airway inflammation and protect the structure of AEB.

Glucocorticoids modulate inflammation through multiple mechanisms: they enhance the secretion of anti‐inflammatory mediators while suppressing the release and activity of pro‐inflammatory factors. Glucocorticoids can promote the secretion of mitogen‐activated protein kinase (MAPK) phosphatases, thereby inhibiting the MAPK signaling pathway and exerting inhibitory effects on downstream inflammatory responses [32]. Additionally, via histone deacetylases (HDACs), glucocorticoids promote the packaging of DNA into transcriptionally inactive chromatin structures, thereby repressing inflammatory gene transcription [33]. They also post‐transcriptionally regulate proinflammatory genes by reversing the stabilization of unstable proinflammatory messenger RNA (mRNA) by inflammatory mediators and accelerating the degradation of them [34, 35]. Furthermore, glucocorticoids reduce the production of asthma‐related interleukins such as IL‐4, IL‐5, and IL‐13, thereby alleviating airway inflammation [36, 37].

Glucocorticoids also inhibit inflammatory cell infiltration through various means. They reduce the migration of inflammatory cells from the circulation to sites of inflammation, partly by interfering with the interactions between cell surface receptors and vascular endothelium [38]. Glucocorticoids influence various cells and mediators involved in the airway vascular function and hyperresponsiveness [39]. They reduce airway inflammatory cell infiltration by decreasing the level of inflammatory cell recruitment factors [36, 37]. In addition, direct inhibitory effects of glucocorticoids are exerted on multiple cell types involved in asthma inflammation, including eosinophils, mast cells, airway smooth muscle cells, epithelial cells, macrophages, and T lymphocytes [40].

Eosinophils are particularly sensitive to glucocorticoids in asthmatic airways. Glucocorticoids alleviate airway eosinophilic inflammation by altering eosinophil distribution and inducing apoptosis. Within hours of administration, glucocorticoids can induce eosinophil migration to the bone marrow through C‐X‐C chemokine receptor type 4 (CXCR4)‐dependent mechanisms, thereby reducing the number of peripheral blood and airway eosinophils [41]. Glucocorticoids also diminish cytokine‐mediated eosinophil survival and directly promote eosinophil apoptosis via HDAC dependent pathways [42, 43]. Through these mechanisms, glucocorticoids exert potent anti‐inflammatory effects in asthma, inhibiting airway inflammatory cell infiltration and mitigating AEB impairment caused by these inflammatory cells.

Beyond indirectly protecting the AEB via inflammation suppression, glucocorticoids have been shown to directly promote AEB integrity, thereby reducing the invasion and sensitization of allergens and pathogens [44]. Glucocorticoids inhibit goblet cell hyperplasia and mucus hypersecretion in asthmatic airways, helping to maintain a normal airway surface liquid layer and reducing mucus plugging and pathogen accumulation [45]. They also maintain the normal structure of airway and inhibit airway remodeling [45].

## 6 Dual Effects of Glucocorticoids on the Airway Epithelial Barrier: Protection and Potential Impairment

Despite their well‐established protective role, emerging evidence paradoxically suggests that glucocorticoids may also undermine the AEB they are meant to protect. Glucocorticoid administration may induce airway epithelial cell apoptosis, thereby disrupting the integrity of the AEB and increasing exposure to allergens and pathogens [46]. In vitro studies have demonstrated that glucocorticoids can trigger the apoptosis and inhibit the proliferation of airway epithelial cells, potentially inhibiting the repair of impaired AEB [47, 48].

These findings are supported by animal studies. In models using asthmatic mice and rabbits, glucocorticoid treatment increased epithelial cell injury, apoptosis, and shedding, leading to impaired barrier function and elevated epithelial cell counts in bronchoalveolar lavage fluid [49, 50, 51]. Additionally, Yilmaz et al. reported that glucocorticoid administration reduced airway epithelial thickness in a murine asthma model, which may weaken its airway barrier function against pathogens and allergens [45].

This paradoxical damaging potential raises important questions about its contribution to glucocorticoid resistance.

This duality presents a clinical and pathophysiological paradox: How can a cornerstone therapy both protect and impair the same structure? Resolving this paradox may be critical to understanding glucocorticoid treatment failure.

## 7 Glucocorticoid Resistance in Some Children With Asthma and Its Link to Severe Asthma

Most asthma patients respond well to ICS, with improved lung function and fewer asthma exacerbations. However, 5%–10% of asthma patients exhibit a suboptimal response to glucocorticoid therapy, leading to poorly controlled symptoms [4]. Among them, those refractory asthmas excluding cases caused by poor medication adherence or inadequate inhalation technique are termed severe asthma [4]. The underlying mechanisms of severe asthma are multifactorial and not fully elucidated, but glucocorticoid resistance is likely a key contributor.

Studies have found that in certain cases, glucocorticoids fail to adequately reduce airway inflammation and lower pro‐inflammatory cytokine levels or suppress inflammatory cell infiltration. For example, dexamethasone treatment sometimes fails to normalize the levels of IL‐1β, IL‐5, and IL‐17 in the asthmatic airway and was even reported to upregulate these proinflammatory cytokines in cellular studies [52, 53, 54, 55]. Other studies show glucocorticoids can induce the expression of various inflammation‐related genes and promote release of pro‐inflammatory molecules [56, 57]. As for the airway inflammatory cell levels, some studies have found that glucocorticoids fail to reduce airway inflammatory cell infiltration [58]. The reasons for this diminished efficacy are unclear but may be closely related to AEB impairment.

## 8 Airway Epithelial Barrier Impairment Is a Potential Contributor to Glucocorticoid Resistance and Severe Asthma

AEB dysfunction may be a key factor in the development of glucocorticoid resistance and severe asthma. Proposed mechanisms include increased release of pro‐inflammatory chemokines, enhanced invasion and sensitization by pathogens and allergens, and other consequences of AEB impairment.

As noted, airway epithelial cells are central to regulating inflammatory mediators in asthmatic airway. Their injury, apoptosis, and shedding can directly release various chemokines and a large number of endogenous antigens, which will activate various inflammatory cells and lead to the activation of the inflammatory cascade. Overexpression of various immune and inflammatory mediators, such as IL‐2, IL‐4, and IL‐13, is involved in the development of glucocorticoid resistance and severe asthma [59, 60, 61].

Inflammatory dysregulation caused by the overexpression of IL‐2, IL‐4, and IL‐13 may interfere with glucocorticoid signaling pathways, leading to glucocorticoid resistance [62, 63]. Elevated expression of these cytokines has been shown to be associated with reduced GR affinity, with a mechanism involving the activation of p38 MAPK, which induces GR phosphorylation and decreases its nuclear translocation in inflammatory cells [64]. Furthermore, Th1 cytokines may also lower glucocorticoid sensitivity in human airway epithelium. For example, tumor necrosis factor alpha (TNF‐α) and IFN‐γ can maintain glucocorticoid resistance in airway smooth muscle cells through multiple mechanisms [65], and IL‐17A reduces the glucocorticoid sensitivity of airway epithelium [66]. IFN‐γ can upregulate the expression of miR‐9 in the airway and pulmonary macrophages, increasing GR phosphorylation and inhibiting its nuclear translocation [67].

Moreover, impaired physical and chemical barrier function facilitates the entry of pathogens and allergens. Infections significantly impact asthma control and contribute to glucocorticoid resistance and severe asthma. Glucocorticoids can inhibit innate and adaptive antiviral immune responses in the airway, delaying viral clearance and increasing mucus production, reducing antimicrobial peptide secretion, and raising bacterial loads—all these factors worsen the disease [68, 69, 70].

Various pathogens, including *Chlamydia pneumonia*, *Haemophilus influenzae*, rhinovirus, influenza A virus (IAV), and respiratory syncytial virus (RSV), can reduce glucocorticoid sensitivity through distinct signaling pathways, thereby inducing glucocorticoid resistance in asthma [71, 72, 73, 74, 75]. For example, in rhinovirus‐infected human bronchial epithelial cells, the activation of nuclear factor κB (NF‐κB) and c‐Jun N‐terminal kinase (JNK) reduces GR‐α nuclear translocation, a mechanism underlying glucocorticoid insensitivity [75]. Certain bacterial products can induce glucocorticoid resistance associated with bacterial infections [76]. For fungal‐related glucocorticoid resistance, mechanisms may involve the induction of Th2/Th17 responses [77].

## 9 AEB Protection for Children With Asthma Requires More Attention

The vulnerability of the AEB in children is not merely a matter of anatomic immaturity but is embedded in the dynamic processes of postnatal lung and immune development. During the critical period of postnatal development, the AEB is particularly susceptible to environmental insults (e.g., viral infections and allergens), which can maladaptively program the epithelial function and determine the subsequent glucocorticoid response.

Children have small airway diameters, immature ciliary structure and function, and impaired capacity to clear airway secretions. The development of goblet cells and glands is also incomplete. Furthermore, children are more susceptible to the influence of vitamin A [78] and vitamin D deficiencies [79]. The latter two are of great significance for airway immunity and may be related to the maintenance of AEB [80]. The deficiency of vitamin A in children with asthma is often more severe than that in healthy children. Therefore, we need to pay more attention to protecting the AEB of children.

Children, particularly infants, rely more heavily on innate immunity. Their immune system is not fully developed, and children have more frequent respiratory tract infections than adults. The impairment to the AEB caused by infections is more common in children than in adults. Therefore, the integrity of the AEB is even more important for children with asthma.

Respiratory syncytial virus is a significant pathogen causing respiratory infections in children [81]. It can impair the AEB and trigger airway inflammation, which is closely related to the development of asthma [82]. Moreover, chronic glucocorticoid administration can lead to immunosuppression [83], which may increase the severity of RSV infection. Therefore, investigating whether enhancing AEB protection in children can mitigate infection‐triggered inflammation and asthma inception represents a promising future research direction.

Most of the childhood asthma cases are eosinophilic, which responds better to glucocorticoids than noneosinophilic asthma [84]. However, there are still a large number of cases of childhood asthma that are not correctly diagnosed and treated [85, 86]. More than 80% of asthma cases begin before the age of 3, and the impairment of lung function in children with asthma often begins during the preschool period. Over time, this may lead to airway remodeling and even develop into chronic obstructive pulmonary disease (COPD) in adulthood. Therefore, AEB protection should start already in childhood.

## 10 Future Perspectives and Therapeutic Implications

To translate these insights into improved outcomes, a multi‐pronged agenda for research and clinical practice is needed.

Future research should leverage advanced technologies, including airway organoids and single‐cell and spatial omics, to dynamically resolve the effects of glucocorticoids on pediatric AEB cellular state and intercellular communication.

Furthermore, we may potentially use the identification of AEB integrity biomarkers (such as specific exfoliated epithelial cells and fragments of cell junction proteins) to predict the efficacy of glucocorticoid treatment.

Clinically, integrating AEB functional assessments into asthma management could enable the exploration of more personalized glucocorticoid regimens and adjunctive strategies (such as targeted applications of vitamin A and vitamin D, novel barrier enhancers, and biologics targeting specific epithelial‐derived active factors) in order to minimize potential AEB impairment.

## 11 Conclusion

In summary, the airway epithelial barrier is pivotal in maintaining respiratory homeostasis, and its impairment is central to asthma pathogenesis. In children, the interplay of physiological immaturity, evolving immunity, and nutritional factors confers unique susceptibility to AEB dysfunction. Glucocorticoids, as the cornerstone of asthma therapy, exert multifaceted anti‐inflammatory effects. Yet, accumulating evidence underscores their dual impact on the AEB—a paradox where therapeutic protection coexists with potential barrier impairment. We posit that this duality, particularly when the balance tips toward impairment, may be a fundamental driver of glucocorticoid resistance and the severe asthma phenotype.

Moving forward, elucidating the precise mechanisms of glucocorticoid‐induced AEB impairment and developing strategies to mitigate it—such as leveraging the adjunctive potential of vitamins A and D, enhancing post‐infection (e.g., respiratory syncytial virus) barrier repair, or discovering novel barrier‐strengthening agents—are critical. Simultaneously, advancing tools like airway organoids and single‐cell omics will be essential to dissect the dynamic glucocorticoid‐AEB interplay in the developing lung. Ultimately, integrating AEB integrity assessment into clinical frameworks and adopting a more “barrier‐protecting” approach to glucocorticoid therapy may pave the way for personalized strategies, improving control and potentially reducing the burden of severe, treatment‐resistant asthma in children.

## Author Contributions

**Tangtiaochu Gan:** investigation, formal analysis, writing – original draft, data curation, visualization, project administration. **Chao Niu:** conceptualization, supervision, funding acquisition, writing – review and editing. **Zhou Fu:** supervision, writing – review and editing. All authors are in agreement with the manuscript for publication.

## Ethics Statement

The authors have nothing to report.

## Consent

The authors have nothing to report.

## Conflicts of Interest

Chao Niu is a member of *Pediatric Discovery* Editorial Board. To minimize bias, he was excluded from all editorial decision‐making related to the acceptance of this article for publication. The other authors declare no conflicts of interest.

## Data Availability

The study did not use any original data.

## References

[1] M. I. Asher , C. E. Rutter , K. Bissell , et al., “Worldwide Trends in the Burden of Asthma Symptoms in School‐Aged Children: Global Asthma Network Phase I Cross‐Sectional Study,” Lancet 398, no. 10311 (October 2021): 1569–1580, 10.1016/s0140-6736(21)01450-1. 34755626 PMC8573635

[2] J. Hong and Y. Bao , “Emphasis on Standardized Diagnosis and Treatment of Bronchial Asthma in Children” [in Chinese], Chinese Journal of Pediatrics 54, no. 3 (March 2016): 161–162, 10.3760/cma.j.issn.0578-1310.2016.03.001. 26957059

[3] L. Xiang , J. Zhao , Y. Zheng , et al., “Uncontrolled Asthma and Its Risk Factors in Chinese Children: A Cross‐Sectional Observational Study,” Journal of Asthma 53, no. 7 (September 2016): 699–706, 10.3109/02770903.2016.1144199. 27043467

[4] S. T. Holgate and R. Polosa , “The Mechanisms, Diagnosis, and Management of Severe Asthma in Adults,” Lancet 368, no. 9537 (August 2006): 780–793, 10.1016/S0140-6736(06)69288-X. 16935689

[5] A. Banno , A. T. Reddy , S. P. Lakshmi , and R. C. Reddy , “Bidirectional Interaction of Airway Epithelial Remodeling and Inflammation in Asthma,” Clinical Science 134, no. 9 (May 2020): 1063–1079, 10.1042/CS20191309. 32369100

[6] C. L. Grainge and D. E. Davies , “Epithelial Injury and Repair in Airways Diseases,” Chest 144, no. 6 (December 2013): 1906–1912, 10.1378/chest.12-1944. 24297122

[7] M. Weitnauer , V. Mijošek , and A. H. Dalpke , “Control of Local Immunity by Airway Epithelial Cells,” Mucosal Immunology 9, no. 2 (March 2016): 287–298, 10.1038/mi.2015.126. 26627458

[8] S. T. Gohy , C. Hupin , C. Pilette , and M. Z. Ladjemi , “Chronic Inflammatory Airway Diseases: The Central Role of the Epithelium Revisited,” Clinical and Experimental Allergy 46, no. 4 (April 2016): 529–542, 10.1111/cea.12712. 27021118

[9] B. N. Lambrecht and H. Hammad , “The Airway Epithelium in Asthma,” Nature Medicine 18, no. 5 (May 2012): 684–692, 10.1038/nm.2737. 22561832

[10] D. Parker and A. Prince , “Innate Immunity in the Respiratory Epithelium,” American Journal of Respiratory Cell and Molecular Biology 45, no. 2 (August 2011): 189–201, 10.1165/rcmb.2011-0011RT. 21330463 PMC3175551

[11] R. Bals and P. S. Hiemstra , “Innate Immunity in the Lung: How Epithelial Cells Fight Against Respiratory Pathogens,” European Respiratory Journal 23, no. 2 (February 2004): 327–333, 10.1183/09031936.03.00098803. 14979512

[12] T. S. Hallstrand , T. L. Hackett , W. A. Altemeier , G. Matute‐Bello , P. M. Hansbro , and D. A. Knight , “Airway Epithelial Regulation of Pulmonary Immune Homeostasis and Inflammation,” Clinical Immunology 151, no. 1 (March 2014): 1–15, 10.1016/j.clim.2013.12.003. 24503171

[13] A. Barbato , G. Turato , S. Baraldo , et al., “Epithelial Damage and Angiogenesis in the Airways of Children With Asthma,” American Journal of Respiratory and Critical Care Medicine 174, no. 9 (November 2006): 975–981, 10.1164/rccm.200602-189OC. 16917118

[14] W. Gao , L. Li , Y. Wang , et al., “Bronchial Epithelial Cells: The Key Effector Cells in the Pathogenesis of Chronic Obstructive Pulmonary Disease?,” Respirology 20, no. 5 (July 2015): 722–729, 10.1111/resp.12542. 25868842

[15] K. Hirota , H. Yoshitomi , M. Hashimoto , et al., “Preferential Recruitment of CCR6‐Expressing Th17 Cells to Inflamed Joints via CCL20 in Rheumatoid Arthritis and Its Animal Model,” Journal of Experimental Medicine 204, no. 12 (November 2007): 2803–2812, 10.1084/jem.20071397. 18025126 PMC2118525

[16] J. A. Hirota , M. J. Gold , P. R. Hiebert , et al., “The Nucleotide‐Binding Domain, Leucine‐Rich Repeat Protein 3 Inflammasome/IL‐1 Receptor I Axis Mediates Innate, But Not Adaptive, Immune Responses After Exposure to Particulate Matter Under 10 μm,” American Journal of Respiratory Cell and Molecular Biology 52, no. 1 (January 2015): 96–105, 10.1165/rcmb.2014-0158OC. 24988285

[17] A. Carsin , J. Mazenq , A. Ilstad , J. C. Dubus , P. Chanez , and D. Gras , “Bronchial Epithelium in Children: A Key Player in Asthma,” European Respiratory Review 25, no. 140 (June 2016): 158–169, 10.1183/16000617.0101-2015. 27246593 PMC9487245

[18] A. Kato and R. P. Schleimer , “Beyond Inflammation: Airway Epithelial Cells are at the Interface of Innate and Adaptive Immunity,” Current Opinion in Immunology 19, no. 6 (December 2007): 711–720, 10.1016/j.coi.2007.08.004. 17928212 PMC2196222

[19] A. C. Y. Hsu , K. Parsons , I. Barr , et al., “Critical Role of Constitutive Type I Interferon Response in Bronchial Epithelial Cell to Influenza Infection,” PLoS One 7, no. 3 (2012): e32947, 10.1371/journal.pone.0032947. 22396801 PMC3292582

[20] S. F. Ziegler and D. Artis , “Sensing the Outside World: TSLP Regulates Barrier Immunity,” Nature Immunology 11, no. 4 (April 2010): 289–293, 10.1038/ni.1852. 20300138 PMC2924817

[21] D. Gras , P. Chanez , I. Vachier , A. Petit , and A. Bourdin , “Bronchial Epithelium as a Target for Innovative Treatments in Asthma,” Pharmacology & Therapeutics 140, no. 3 (December 2013): 290–305, 10.1016/j.pharmthera.2013.07.008. 23880290

[22] M. J. Gold , F. Antignano , T. Y. F. Halim , et al., “Group 2 Innate Lymphoid Cells Facilitate Sensitization to Local, But Not Systemic, TH2‐Inducing Allergen Exposures,” Journal of Allergy and Clinical Immunology 133, no. 4 (April 2014): 1142–1148, 10.1016/j.jaci.2014.02.033. 24679471

[23] T. Y. F. Halim , R. H. Krauss , A. C. Sun , and F. Takei , “Lung Natural Helper Cells are a Critical Source of Th2 Cell‐Type Cytokines in Protease Allergen‐Induced Airway Inflammation,” Immunity 36, no. 3 (March 2012): 451–463, 10.1016/j.immuni.2011.12.020. 22425247

[24] S. Montefort , W. R. Roche , and S. T. Holgate , “Bronchial Epithelial Shedding in Asthmatics and Non‐Asthmatics,” supplement, Respiratory Medicine 87, no. sB (August 1993): 9–11, 10.1016/0954-6111(93)90118-j. 8234974

[25] C. L. Ordoñez , R. Khashayar , H. H. Wong , et al., “Mild and Moderate Asthma Is Associated With Airway Goblet Cell Hyperplasia and Abnormalities in Mucin Gene Expression,” American Journal of Respiratory and Critical Care Medicine 163, no. 2 (February 2001): 517–523, 10.1164/ajrccm.163.2.2004039. 11179133

[26] H. A. Jenkins , C. Cool , S. J. Szefler , et al., “Histopathology of Severe Childhood Asthma: A Case Series,” Chest 124, no. 1 (July 2003): 32–41, 10.1378/chest.124.1.32. 12853499

[27] J. Parker , S. Sarlang , S. Thavagnanam , et al., “A 3‐D Well‐Differentiated Model of Pediatric Bronchial Epithelium Demonstrates Unstimulated Morphological Differences Between Asthmatic and Nonasthmatic Cells,” Pediatric Research 67, no. 1 (January 2010): 17–22, 10.1203/PDR.0b013e3181c0b200. 19755931

[28] H. Aegerter and B. N. Lambrecht , “The Pathology of Asthma: What Is Obstructing Our View?,” Annual Review of Pathology: Mechanisms of Disease 18, no. 1 (January 2023): 387–409, 10.1146/annurev-pathol-042220-015902. 36270294

[29] R. Chan , C. Duraikannu , and B. Lipworth , “Clinical Associations of Mucus Plugging in Moderate to Severe Asthma,” Journal of Allergy and Clinical Immunology: In Practice 11, no. 1 (January 2023): 195–199.e2, 10.1016/j.jaip.2022.09.008. 36152990

[30] K. Looi , T. Iosifidis , S. Harrison , et al., “Innate Epithelial and Functional Differences in Airway Epithelium of Children With Acute Wheeze,” Frontiers in Cell and Developmental Biology 13 (2025): 1606915, 10.3389/fcell.2025.1606915. 40791985 PMC12336829

[31] C. Stahn and F. Buttgereit , “Genomic and Nongenomic Effects of Glucocorticoids,” Nature Clinical Practice Rheumatology 4, no. 10 (October 2008): 525–533, 10.1038/ncprheum0898. 18762788

[32] P. J. Barnes , “Glucocorticosteroids,” Handbook of Experimental Pharmacology 237 (2017): 93–115, 10.1007/164_2016_62. 27796513

[33] P. J. Barnes , “Histone Deacetylase‐2 and Airway Disease,” Therapeutic Advances in Respiratory Disease 3, no. 5 (October 2009): 235–243, 10.1177/1753465809348648. 19812111

[34] M. W. Bergmann , K. J. Staples , S. J. Smith , P. J. Barnes , and R. Newton , “Glucocorticoid Inhibition of Granulocyte Macrophage‐Colony‐Stimulating Factor From T Cells Is Independent of Control by Nuclear Factor‐kappaB and Conserved Lymphokine Element 0,” American Journal of Respiratory Cell and Molecular Biology 30, no. 4 (April 2004): 555–563, 10.1165/rcmb.2003-0295oc. 14527927

[35] A. A. Alangari , “Genomic and Non‐Genomic Actions of Glucocorticoids in Asthma,” Annals of Thoracic Medicine 5, no. 3 (July 2010): 133–139, 10.4103/1817-1737.65040. 20835306 PMC2930650

[36] M. Guan , H. Ma , X. Fan , X. Chen , M. Miao , and H. Wu , “Dexamethasone Alleviate Allergic Airway Inflammation in Mice by Inhibiting the Activation of NLRP3 Inflammasome,” International Immunopharmacology 78 (January 2020): 106017, 10.1016/j.intimp.2019.106017. 31780368

[37] R. H. Patil , M. Naveen Kumar , K. M. Kiran Kumar , et al., “Dexamethasone Inhibits Inflammatory Response via Down Regulation of AP‐1 Transcription Factor in Human Lung Epithelial Cells,” Gene 645 (March 2018): 85–94, 10.1016/j.gene.2017.12.024. 29248584

[38] C. G. Westergaard , C. Porsbjerg , and V. Backer , “Emerging Corticosteroid Agonists for the Treatment of Asthma,” Expert Opinion on Emerging Drugs 20, no. 4 (2015): 653–662, 10.1517/14728214.2015.1061503. 26108455

[39] K. Ito , S. J. Getting , and C. E. Charron , “Mode of Glucocorticoid Actions in Airway Disease,” Scientific World Journal 6 (December 2006): 1750–1769, 10.1100/tsw.2006.274. 17195873 PMC5917285

[40] G. Pelaia , A. Vatrella , M. T. Busceti , et al., “Molecular and Cellular Mechanisms Underlying the Therapeutic Effects of Budesonide in Asthma,” Pulmonary Pharmacology & Therapeutics 40 (October 2016), 10.1016/j.pupt.2016.07.001. 27381656

[41] S. G. Hong , N. Sato , F. Legrand , et al., “Glucocorticoid‐Induced Eosinopenia Results From CXCR4‐Dependent Bone Marrow Migration,” Blood 136, no. 23 (December 2020): 2667–2678, 10.1182/blood.2020005161. 32659786 PMC7735160

[42] A. Druilhe , S. Létuvé , and M. Pretolani , “Glucocorticoid‐Induced Apoptosis in Human Eosinophils: Mechanisms of Action,” Apoptosis 8, no. 5 (October 2003): 481–495, 10.1023/a:1025590308147. 12975579

[43] S. Brode , N. Farahi , A. S. Cowburn , J. K. Juss , A. M. Condliffe , and E. R. Chilvers , “Interleukin‐5 Inhibits Glucocorticoid‐Mediated Apoptosis in Human Eosinophils,” Thorax 65, no. 12 (December 2010): 1116–1117, 10.1136/thx.2009.124909. 20805156

[44] C. Rimmer , S. Hetelekides , S. I. Eliseeva , S. N. Georas , and J. M. Veazey , “Budesonide Promotes Airway Epithelial Barrier Integrity Following Double‐Stranded RNA Challenge,” PLoS One 16, no. 12 (2021): e0260706, 10.1371/journal.pone.0260706. 34871316 PMC8648122

[45] O. Yilmaz , M. Karaman , H. A. Bagriyanik , et al., “Comparison of TNF Antagonism by Etanercept and Dexamethasone on Airway Epithelium and Remodeling in an Experimental Model of Asthma,” International Immunopharmacology 17, no. 3 (November 2013): 768–773, 10.1016/j.intimp.2013.08.021. 24063972

[46] D. R. Dorscheid , K. R. Wojcik , S. Sun , B. Marroquin , and S. R. White , “Apoptosis of Airway Epithelial Cells Induced by Corticosteroids,” American Journal of Respiratory and Critical Care Medicine 164, no. 10 Pt 1 (November 2001): 1939–1947, 10.1164/ajrccm.164.10.2103013. 11734450

[47] D. R. Dorscheid , B. J. Patchell , O. Estrada , B. Marroquin , R. Tse , and S. R. White , “Effects of Corticosteroid‐Induced Apoptosis on Airway Epithelial Wound Closure in Vitro,” American Journal of Physiology ‐ Lung Cellular and Molecular Physiology 291, no. 4 (October 2006): L794–L801, 10.1152/ajplung.00322.2005. 16751221

[48] J. Liu , M. Zhang , C. Niu , et al., “Dexamethasone Inhibits Repair of Human Airway Epithelial Cells Mediated by Glucocorticoid‐Induced Leucine Zipper (GILZ),” PLoS One 8, no. 4 (April 2013): e60705, 10.1371/journal.pone.0060705. 23573276 PMC3615997

[49] D. R. Dorscheid , E. Low , A. Conforti , S. Shifrin , A. I. Sperling , and S. R. White , “Corticosteroid‐Induced Apoptosis in Mouse Airway Epithelium: Effect in Normal Airways and After Allergen‐Induced Airway Inflammation,” Journal of Allergy and Clinical Immunology 111, no. 2 (February 2003): 360–366, 10.1067/mai.2003.117. 12589357

[50] J. Uhlík , L. Vajner , J. Adásková , and V. Konrádová , “Effect of Inhalation of Single Dose of Beclomethasone on Airway Epithelium,” Ultrastructural Pathology 31, no. 3 (2007): 221–232, 10.1080/01913120701425951. 17614001

[51] C. Niu , T. Wang , W. Zou , et al., “Enhanced Pause Correlates With Airway Neutrophils and Airway‐Epithelial Injury in Asthmatic Mice Treated With Dexamethasone,” Journal of Asthma 56, no. 1 (January 2019): 11–20, 10.1080/02770903.2018.1494190. 29985082

[52] A. Gupta , S. Dimeloe , D. F. Richards , et al., “Defective IL‐10 Expression and in Vitro Steroid‐Induced IL‐17A in Paediatric Severe Therapy‐Resistant Asthma,” Thorax 69, no. 6 (June 2014): 508–515, 10.1136/thoraxjnl-2013-203421. 24347461

[53] A. M. Nanzer , E. S. Chambers , K. Ryanna , et al., “Enhanced Production of IL‐17A in Patients With Severe Asthma Is Inhibited by 1α,25‐Dihydroxyvitamin D3 in a Glucocorticoid‐Independent Fashion,” Journal of Allergy and Clinical Immunology 132, no. 2 (August 2013): 297–304.e3, 10.1016/j.jaci.2013.03.037. 23683514

[54] G. Caramori , F. Nucera , S. Mumby , F. Lo Bello , and I. M. Adcock , “Corticosteroid Resistance in Asthma: Cellular and Molecular Mechanisms,” Molecular Aspects of Medicine 85 (June 2022): 100969, 10.1016/j.mam.2021.100969. 34090658

[55] M. Kaur , S. Reynolds , L. J. Smyth , K. Simpson , S. Hall , and D. Singh , “The Effects of Corticosteroids on Cytokine Production From Asthma Lung Lymphocytes,” International Immunopharmacology 23, no. 2 (December 2014): 581–584, 10.1016/j.intimp.2014.10.008. 25466265

[56] A. Bansal , C. Kooi , K. Kalyanaraman , et al., “Synergy Between Interleukin‐1β, Interferon‐γ, and Glucocorticoids to Induce TLR2 Expression Involves NF‐κB, STAT1, and the Glucocorticoid Receptor,” Molecular Pharmacology 105, no. 1 (December 2023): 23–38, 10.1124/molpharm.123.000740. 37863662

[57] J. M. Busillo , K. M. Azzam , and J. A. Cidlowski , “Glucocorticoids Sensitize the Innate Immune System Through Regulation of the NLRP3 Inflammasome,” Journal of Biological Chemistry 286, no. 44 (November 2011): 38703–38713, 10.1074/jbc.M111.275370. 21940629 PMC3207479

[58] D. C. Cowan , J. O. Cowan , R. Palmay , A. Williamson , and D. R. Taylor , “Effects of Steroid Therapy on Inflammatory Cell Subtypes in Asthma,” Thorax 65, no. 5 (May 2010): 384–390, 10.1136/thx.2009.126722. 19996343

[59] N. C. Nicolaides and E. Charmandari , “Novel Insights Into the Molecular Mechanisms Underlying Generalized Glucocorticoid Resistance and Hypersensitivity Syndromes,” Hormones 16, no. 2 (April 2017): 124–138, 10.14310/horm.2002.1728. 28742501

[60] O. Keskin , Ü Uluca , E. Birben , et al., “Genetic Associations of the Response to Inhaled Corticosteroids in Children During an Asthma Exacerbation,” Pediatric Allergy & Immunology 27, no. 5 (August 2016): 507–513, 10.1111/pai.12566. 27003716

[61] M. Rijavec , M. Žavbi , A. Lopert , M. Fležar , and P. Korošec , “GLCCI1 Polymorphism rs37973 and Response to Treatment of Asthma With Inhaled Corticosteroids,” Journal of Investigational Allergology & Clinical Immunology 28, no. 3 (2018): 165–171, 10.18176/jiaci.0229. 29345236

[62] D. Gurgone , L. McShane , C. McSharry , T. J. Guzik , and P. Maffia , “Cytokines at the Interplay Between Asthma and Atherosclerosis?,” Frontiers in Pharmacology 11 (2020): 166, 10.3389/fphar.2020.00166. 32194407 PMC7064545

[63] M. C. Peters and S. E. Wenzel , “Intersection of Biology and Therapeutics: Type 2 Targeted Therapeutics for Adult Asthma,” Lancet 395, no. 10221 (February 2020): 371–383, 10.1016/S0140-6736(19)33005-3. 32007172 PMC8522504

[64] K. Ito , S. Yamamura , S. Essifie‐Quaye , et al., “Histone Deacetylase 2‐Mediated Deacetylation of the Glucocorticoid Receptor Enables NF‐kappaB Suppression,” Journal of Experimental Medicine 203, no. 1 (January 2006), 10.1084/jem.20050466. PMC2118081 16380507

[65] R. D. Britt , M. A. Thompson , S. Sasse , C. M. Pabelick , A. N. Gerber , and Y. S. Prakash , “Th1 Cytokines TNF‐α and IFN‐γ Promote Corticosteroid Resistance in Developing Human Airway Smooth Muscle,” American Journal of Physiology ‐ Lung Cellular and Molecular Physiology 316, no. 1 (January 2019): L71–L81, 10.1152/ajplung.00547.2017. 30335498 PMC6383501

[66] S. F. Rahmawati , R. Vos , I. S. T. Bos , H. A. M. Kerstjens , L. E. M. Kistemaker , and R. Gosens , “Function‐Specific IL‐17A and Dexamethasone Interactions in Primary Human Airway Epithelial Cells,” Scientific Reports 12, no. 1 (June 2022): 11110, 10.1038/s41598-022-15393-2. 35773318 PMC9247091

[67] J. J. Li , H. L. Tay , S. Maltby , et al., “MicroRNA‐9 Regulates Steroid‐Resistant Airway Hyperresponsiveness by Reducing Protein Phosphatase 2A Activity,” Journal of Allergy and Clinical Immunology 136, no. 2 (August 2015): 462–473, 10.1016/j.jaci.2014.11.044. 25772595

[68] A. Singanayagam , N. Glanville , J. L. Girkin , et al., “Corticosteroid Suppression of Antiviral Immunity Increases Bacterial Loads and Mucus Production in COPD Exacerbations,” Nature Communications 9, no. 1 (June 2018): 2229, 10.1038/s41467-018-04574-1. PMC5993715 29884817

[69] A. Marcellini , D. Swieboda , A. Guedán , et al., “Glucocorticoids Impair Type I IFN Signalling and Enhance Rhinovirus Replication,” European Journal of Pharmacology 893 (February 2021): 173839, 10.1016/j.ejphar.2020.173839. 33359650

[70] P. Wang , X. Wang , X. Yang , Z. Liu , M. Wu , and G. Li , “Budesonide Suppresses Pulmonary Antibacterial Host Defense by Down‐Regulating Cathelicidin‐Related Antimicrobial Peptide in Allergic Inflammation Mice and in Lung Epithelial Cells,” BMC Immunology 14, no. 1 (February 2013): 7, 10.1186/1471-2172-14-7. 23387852 PMC3583690

[71] A. T. Essilfie , J. L. Simpson , J. C. Horvat , et al., “ *Haemophilus Influenzae* Infection Drives IL‐17‐Mediated Neutrophilic Allergic Airways Disease,” PLoS Pathogens 7, no. 10 (October 2011): e1002244, 10.1371/journal.ppat.1002244. 21998577 PMC3188527

[72] J. Beale , A. Jayaraman , D. J. Jackson , et al., “Rhinovirus‐Induced IL‐25 in Asthma Exacerbation Drives Type 2 Immunity and Allergic Pulmonary Inflammation,” Science Translational Medicine 6, no. 256 (October 2014): 256ra134, 10.1126/scitranslmed.3009124. PMC4246061 25273095

[73] X. Yang , Y. Wang , S. Zhao , R. Wang , and C. Wang , “Long‐Term Exposure to Low‐Dose *Haemophilus Influenzae* During Allergic Airway Disease Drives a Steroid‐Resistant Neutrophilic Inflammation and Promotes Airway Remodeling,” Oncotarget 9, no. 38 (May 2018): 24898–24913, 10.18632/oncotarget.24653. 29861841 PMC5982741

[74] D. Paróczai , T. Mosolygó , D. Kókai , et al., “ *Chlamydia Pneumoniae* Influence on Cytokine Production in Steroid‐Resistant and Steroid‐Sensitive Asthmatics,” Pathogens 9, no. 2 (February 2020): 112, 10.3390/pathogens9020112. 32054098 PMC7167821

[75] A. Papi , M. Contoli , I. M. Adcock , et al., “Rhinovirus Infection Causes Steroid Resistance in Airway Epithelium Through Nuclear Factor κB and c‐Jun N‐terminal Kinase Activation,” Journal of Allergy and Clinical Immunology 132, no. 5 (November 2013): 1075–1085.e6, 10.1016/j.jaci.2013.05.028. 23871663

[76] J. Reidl and E. Monsó , “Glucocorticoids and Antibiotics, How Do They Get Together?,” EMBO Molecular Medicine 7, no. 8 (August 2015): 992–993, 10.15252/emmm.201505336. 26077592 PMC4551337

[77] R. Y. Kim , J. W. Pinkerton , A. T. Essilfie , et al., “Role for NLRP3 Inflammasome‐Mediated, IL‐1β‐Dependent Responses in Severe, Steroid‐Resistant Asthma,” American Journal of Respiratory and Critical Care Medicine 196, no. 3 (August 2017): 283–297, 10.1164/rccm.201609-1830OC. 28252317

[78] S. Castanhinha , R. Sherburn , S. Walker , et al., “Pediatric Severe Asthma With Fungal Sensitization Is Mediated by Steroid‐Resistant IL‐33,” Journal of Allergy and Clinical Immunology 136, no. 2 (August 2015): 312–322.e7, 10.1016/j.jaci.2015.01.016. 25746970 PMC4534777

[79] J. Hu , J. Sang , F. Hao , and L. Liu , “Association Between Vitamin A and Asthma: A Meta‐Analysis With Trial Sequential Analysis,” Frontiers in Pharmacology 14 (2023): 1100002, 10.3389/fphar.2023.1100002. 36794278 PMC9922757

[80] N. Brustad and B. Chawes , “Vitamin D Primary Prevention of Respiratory Infections and Asthma in Early Childhood: Evidence and Mechanisms,” Journal of Allergy and Clinical Immunology 12, no. 7 (July 2024): 1707–1714, 10.1016/j.jaip.2024.02.005. 38360214

[81] E. M. Hollams , N. H. de Klerk , P. G. Holt , and P. D. Sly , “Persistent Effects of Maternal Smoking During Pregnancy on Lung Function and Asthma in Adolescents,” American Journal of Respiratory and Critical Care Medicine 189, no. 4 (February 2014): 401–407, 10.1164/rccm.201302-0323OC. 24251622

[82] F. D. Gilliland , Y. F. Li , and J. M. Peters , “Effects of Maternal Smoking During Pregnancy and Environmental Tobacco Smoke on Asthma and Wheezing in Children,” American Journal of Respiratory and Critical Care Medicine 163, no. 2 (February 2001): 429–436, 10.1164/ajrccm.163.2.2006009. 11179118

[83] J. Elliot , N. Carroll , M. Bosco , M. McCrohan , and P. Robinson , “Increased Airway Responsiveness and Decreased Alveolar Attachment Points Following in Utero Smoke Exposure in the Guinea Pig,” American Journal of Respiratory and Critical Care Medicine 163, no. 1 (January 2001): 140–144, 10.1164/ajrccm.163.1.9805099. 11208639

[84] G. Chatziparasidis , A. Bush , M. R. Chatziparasidi , and A. Kantar , “Airway Epithelial Development and Function: A Key Player in Asthma Pathogenesis?,” Paediatric Respiratory Reviews 1, no. 47 (September 2023): 51–61, 10.1016/j.prrv.2023.04.005. 37330410

[85] D. G. Peroni , K. Hufnagl , P. Comberiati , and F. Roth‐Walter , “Lack of Iron, Zinc, and Vitamins as a Contributor to the Etiology of Atopic Diseases,” Frontiers in Nutrition 9 (2022): 1032481, 10.3389/fnut.2022.1032481. 36698466 PMC9869175

[86] C. Niu , N. Liu , J. Liu , et al., “Vitamin A Maintains the Airway Epithelium in a Murine Model of Asthma by Suppressing Glucocorticoid‐Induced Leucine Zipper,” Clinical and Experimental Allergy 46, no. 6 (June 2016): 848–860, 10.1111/cea.12646. 26399569

